# Skin cancer special edition

**DOI:** 10.1002/ski2.138

**Published:** 2022-06-06

**Authors:** George W. M. Millington

**Affiliations:** ^1^ Dermatology Department Norwich UK

Skin cancer is an increasing challenge globally. Keratinocyte cancers (basal cell carcinoma ‐BCC and squamous cell carcinoma–cSCC) are the commonest cancers in white populations, with rates increasing worldwide.^1^ On average, one in five people in England will develop one of these tumours in their lifetimes.^1^ BCC and cSCC are both more common in males than females in England.^1^ However, BCC and cSCC are both more common in females than males in England under the age of 50.^1^ Melanoma is the 19th most common cancer internationally.^2^ The incidence of melanoma has been increasing worldwide over the past 50 years, especially in white populations and people living nearer to the equator.^2^ Australia and New Zealand have experienced the greatest incidence of melanoma, then North America, as well as the European countries, between 2013 and 2015.^2^ The incidence rates of Merkel cell carcinoma (MCC), an aggressive cutaneous cancer, have tripled from 0.15 cases per 100,000 individuals in 1986 to 0.7 per 100,000 in 2013.^3^ MCC is three times more lethal than melanoma.^3^ Although much of the research into skin cancer is in white populations (skin types I and II), there is a growing realization of skin cancer problems in skin of colour,^4^ Considering all of this, the editorial team of SHD will launch a themed issue early next year on the subject of Cutaneous Oncology. I am extremely grateful to Ewan Langan (Berlin), Oriol Yelamos (Barcelona), Shamir Geller (Tel Aviv) and Selin Tokez (Rotterdam) for agreeing to be guest editors. If you wish to discuss a skin cancer submission with me, or with any of the guest editors, please email shd@bad.org.uk.

## AUTHOR CONTRIBUTIONS

**George W. M. Millington:** Writing – original draft (lead); Writing – review & editing (lead).

## CONFLICT OF INTEREST

George W. M. Millington is the Editor in Chief of Skin Health and Disease.

## Data Availability

Data sharing is not applicable to this article as no new data were created or analyzed in this study.
